# Liquid-type plasma-controlled in situ crosslinking of silk-alginate injectable gel displayed better bioactivities and mechanical properties

**DOI:** 10.1016/j.mtbio.2022.100321

**Published:** 2022-06-10

**Authors:** Sungryeal Kim, Hye-Young Lee, Hye Ran Lee, Jeon Yeob Jang, Ju Hyun Yun, Yoo Seob Shin, Chul-Ho Kim

**Affiliations:** a Department of Otolaryngology, College of Medicine, Inha University, Incheon, South Korea; b Department of Medical Sciences, Graduate School of Ajou University, Suwon, South Korea; c Department of Otolaryngology, School of Medicine, Ajou University, Suwon, South Korea; d Department of Otorhino-laryngology-Head and Neck Surgery, Catholic Kwandong University, College of Medicine, Incheon, South Korea

**Keywords:** Crossliniking, Liquid-type non-thermal atmospheric plasma, Injectable hydrogel, Silk, Wound healing

## Abstract

Silk is a promising biomaterial for injectable hydrogel, but its long-gelation time and cytotoxic crosslinking methods are the main obstacles for clinical application. Here, we purpose a new *in situ* crosslinking technique of silk-alginate (S-A) injectable hydrogel using liquid-type non-thermal atmospheric plasma (LTP) in vocal fold (VF) wound healing. We confirmed that LTP induces the secondary structure of silk in a dose-dependent manner, resulting in improved mechanical properties. Significantly increased crosslinking of silk was observed with reduced gelation time. Moreover, controlled release of nitrate, an LTP effectors, from LTP-treated S-A hydrogel was detected over 7 days. *In vitro* experiments regarding biocompatibility showed activation of fibroblasts beyond the non-cytotoxicity of LTP-treated S-A hydrogels. An *in vivo* animal model of VF injury was established in New Zealand White rabbits. Full-thickness injury was created on the VF followed by hydrogel injection. In histologic analyses, LTP-treated S-A hydrogels significantly reduced a scar formation and promoted favorable wound healing. Functional analysis using videokymography showed eventual viscoelastic recovery. The LTP not only changes the mechanical structures of a hydrogel, but also has sustained biochemical effects on the damaged tissue due to controlled release of LTP effectors, and that LTP-treated S-A hydrogel can be used to enhance wound healing after VF injury.

## Graphical abstract

**Figure:**
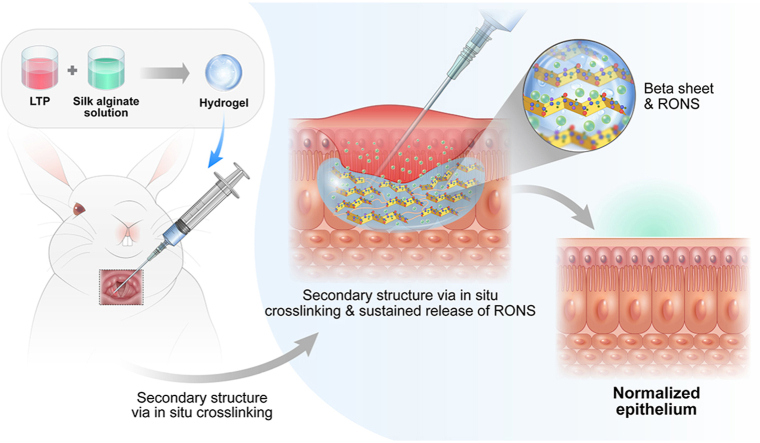

## 1 Introduction

In conventional regenerative medicine, biomaterials can be produced in forms of fiber, 3D structures, films, aerogels, and hydrogels depending on the end use [1]. Among scaffolds, hydrogels are being studied for use as injectable tissue fillers, microscale particle and bio-inks due to tunable physical, chemical and mechanical properties [2]. The injectable hydrogels also have some medical advantages including implantation with minimal incisions and mixing with bioactive component such as drugs and cells [3]. The injectable hydrogel can be made of synthetic polymers or/and natural polymers including silk, alginate, and chitosan. Compared with synthetic polymers, natural polymers have properties especially useful for biomedical applications due to their inherent advantages such as biocompatibility, ease of large-scale production, and facile manipulation [4]. Silk is one of the most promising natural protein polymers for injectable hydrogel due to its advantages of biocompatibility, material versatility and mechanical strength [1,5,6]. Alginate is a natural polymer with high swelling capability, and has been studied in the form of silk-alginate composite hydrogel to improve the performance and expand the range of use of hydrogel [7].

There are two crosslinking preparation methods of silk-based hydrogel, physical and chemical. Self-assembly, a method of physical crosslinking, occurs when silk fibroin solution is kept at room temperature. A conformational change from a random coil to a β-sheet occurs due to thermodynamic instability of the solution, and a hydrogel is created by self-assembly [8,9]. Formation of a hydrogel through self-assembly takes several days, so various crosslinking methods such as physical (temperature, acidity, vortexing, ultrasonication) and chemical (glutaraldehyde, genipin, photocrosslinking, enzyme) are used to reduce preparation time and to control its properties [8]. However, each crosslinking method has limitations. Physical crosslinking is safe and takes less time than self-assembly but still requires several hours [10]. The chemical crosslinking method can be said to be an effective and needs only minutes to produce a hydrogel. However, it uses a cytotoxic chemical solvent and can be of safety concern for medical use [11]. Enzymatic crosslinking using horseradish peroxidase and hydrogen peroxide has recently attracted attention as it can control mechanical strength without toxic materials. However, this process is not suitable for injectable hydrogel due to limitations including relatively slow and weak crosslinking and low cellular attachment of the hydrogel [2]. Research aims to find a suitable crosslinking method for injectable hydrogel preparation [12].

Plasma is the fourth state of matter along with solid, liquid, and gas and is created by adding energy to gas. Historically, plasma was a thermal material with a high temperature exceeding 40 ​°C, limiting its application range. Development of technologies has produced plasma at a temperature of 40 ​°C or less, which is being utilized in various fields such as electrical switching, material synthesis, material processing, esterification, and medical applications [13,14]. Reactive oxygen and nitrogen species (RONS), radiation (ultraviolet, visual light, near-infrared radiation), and electronic fields generated by non-thermal plasma are effective in disinfection, dental care, skin disease, chronic wounds, cosmetics, tissue regeneration, and cancer therapy [15]. Traditional surface modification using wet chemical techniques has environmental and process hazards due to use of large quantities of chemicals and time-consuming steps [16,17]. In comparison, plasma treatment does not require toxic chemicals and is relatively simple. The large atoms, ions, neutrons, and electrons of plasma can participate in many chemical reactions for coatings, etching, and activation of materials [18]. Plasma treatment on biomaterials provides several benefits including (1) enhancing biocompatibility and antibacterial properties, (2) functionalization and etching, (3) hydrophilicity and hydrophobicity control, and (4) modification of mechanical properties [19]. For example, plasma application on silk fibers improved their strength and hydrophobicity [20]. In addition, as plasma can cause crosslinking of proteins and polysaccharides, it is considered as a novel tool to increase the regenerative potential of scaffolds including hydrogels [17,21,22].

Furthermore, plasma can be used either directly or indirectly. The indirect application, known as liquid-type plasma (LTP), is being actively studied in hydrogels because it has strengths in terms of transformation and manipulation [[23], [24], [25]]. An LTP system, which is formed and propagated by electrical discharges in different organic solvents, can be applied as a liquid, is easily mixed with injectable hydrogels, and can modify physical and chemical properties of materials and function biologically after being injected into a patient. Thus, we hypothesized that LTP treatment to a silk-alginate (S–A) hydrogel could control the mechanical properties and enhance the regenerative power. In addition, LTP can not only change the structures of a hydrogel, but also can affect the damaged tissue biochemically in a sustained manner due to controlled release of RONS from S-A hydrogel. In the present study, microstructural analysis and rheology testing of hydrogels were performed according to LTP treatment. Changes in cell viability/mobility/morphology and extracellular matrix components were observed in an *in vitro* model. *In vivo* experiments using mice and rabbits were conducted to determine whether LTP had a regenerative effect in tissue. The aim of this study was to evaluate the potential of LTP as a novel method for *in situ* crosslinking of silk and injectable biomaterial for repair of vocal fold (VF) injury.

## 2 Materials and methods

### 2.1 Chemicals and instruments

Bombyx mori cocoons were purchased from Chunzam Bio in Jeonju, Korea. Water used for all experiments was ultrapure water by Purist Ultrapure Water Systems (resistivity 18.2 ​MΩ ​cm at 25 ​°C, Rephile, China). All chemicals were purchased from Sigma-Aldrich (St Louis, MO, USA).

### 2.2 Preparation of silk-alginate composite hydrogel

Bombyx mori cocoons were cut into small pieces and treated 3 times with boiling distilled water (DW) containing 0.02 ​M sodium carbonate for 30 ​min. The resulting fibers were repeatedly washed with distilled water to remove the sericin protein and were dried in a hot-air oven. Silk fiber mass was dissolved in 9.3 ​M LiBr solution at 70 ​°C for 4 ​h. The dissolved silk fibroin solution was dialyzed against DW for 2 days using SnakeSkinTM 3.5 ​K molecular weight cutoff Dialysis Tubing (Thermo Scientific™, Waltham, MA, USA). Then, the tubing was removed and centrifuged to remove small contaminants. The final silk fibroin solution was stored in a refrigerator until use. The final concentration of silk fibroin solution was 8–10 ​wt% and it was stored in a refrigerator until use. A 1% (w/v) alginate powder was dissolved homogeneously in concentrated silk fibroin solution and autoclaved at 121 ​°C for 5 ​min [[26], [27], [28]]. Three groups of solutions were prepared as follows: (1) alginate, (2) silk-alginate, and (3) LTP-treated silk-alginate, and all three solutions were mixed with calcium chloride solution (250 ​mM) as crosslinker (Table S1).

To prepare LTP-treated silk-alginate hydrogel, the silk-alginate solution was treated by a non-thermal atmospheric dielectric barrier discharge (DBD) plasma spray-type generator (PSM, Korea) supplied with helium and oxygen (15% helium/85% oxygen) mixture as carrier gas. The silk-alginate solution was exposed to He/O_2_ plasma at an input voltage 5 ​kV, operation frequency 25 ​kHz, and gas flow rate 10 standard liters per minute (slm). The plasma was ejected at a distance of 20 ​mm from the liquid surface for 10, 30, 60, and 180 ​s/mL.

### 2.3 Characterization

For surface morphology and microstructure observation, hydrogels were frozen at −80 ​°C, and freeze-dried samples were sputter coated with gold thin film for 210 ​s (Cressington 108 Auto; Ted Pella, Inc., USA). The microstructure of the hydrogel was analyzed with a field emission scanning electron microscope (FE-SEM, JSM-7900 ​F, JEOL, Tokyo, Japan) operated at an acceleration voltage of 3 ​kV. The pore size was determined by selecting 100 pores from each hydrogel and measuring the diameter of the SEM image with ImageJ software.

Fourier-transform infrared (FTIR) spectrometry in the range 4000–400 ​cm^−1^ was performed on a Nicolet iS50 (Thermo Fisher Scientific, USA) instrument. Each measurement was an average of 32 interferograms at a mirror speed of 5.0 ​cm/s.

### 2.4 Swelling test

To calculate the water absorption capacity of freeze-dried hydrogel, the samples were immersed in PBS to different swelling ratios (SRs). SR was calculated using the following equation:

(1) $$Swelling\;ratio\;\left(SR\right)=\frac{Weight\;\left(wet\right)-Weight\;\left(Dry\right)}{Weight\;\left(Dry\right)}$$

where Weight(wet) and Weight(dry) represent the weights of the wet, swollen sample and the initial dry sample, respectively. The swelling test was performed in quadruplicate.

### 2.5 Gelation time and injectability test

The sol gel transition of the hydrogel was determined using the vial-tilting method. Glass vials containing 1 ​mL of hydrogel solution and 250 ​mL CaCl_2_ were inverted every 10s at 25 ​°C. Gelation was confirmed by lack of flow with tilting. To evaluate injectability, a calcium chloride loaded 25G metal-type spinal needle (Hakko, Japan) was combined in a 1 ​mL luer-lock syringe filled with a hydrogel solution, followed by manual application of slow shear force on the plunger. Qualitative evaluation of injectability was performed by visually observing the flow through the needle. This process was repeated three times.

### 2.6 Rheological measurements

Rheological analyses were used for quantifying elastic modulus and complex viscosity of the silk-alginate hydrogels on ARES-G2 (TA Instruments™, USA) with a parallel plate (25 ​mm in diameter with 1 ​mm gap) at 25 ​°C. The hydrogel samples were placed on the bottom plate, and the pressure of the top plate was lowered to 0.1 ​N. Rheological measurements were obtained over a frequency range of 0.1–100 ​rad/s at a fixed deformation strain of 1% to study the viscoelastic properties.

### 2.7 Nitrite determination by griess reagent

The NO release from plasma-treated hydrogel was measured by Griess assay as previously described. Briefly, the hydrogels were immerged in DW and kept in an incubator at 37 ​°C. At each designated time point, a sample of supernatant was mixed with an equal volume of Griess reagent (1 w/v% sulfanilamide, 0.1 w/v% N-(1-naphtyl)ethylenediamine, and 5 w/v% of phosphoric acid in DW). The mixtures were incubated for 10 ​min in darkness at 25 ​°C. The absorbance at 540 ​nm was measured using a microplate reader (BioTek Instruments, USA). The NaNO_2_ standard curve was used to determine the molar amount of nitric oxide released from the supernatant. Each hydrogel group were analyzed in triplicate and the data are shown as mean ​± ​standard deviation.

### 2.8 Immunocytochemistry

Cell adhesion and localization of FAK (3285, Cell Signaling, 1:500) and YAP/TAZ (8418, Cell Signaling, 1:500) were examined with hVFFs. Cells were seeded onto hydrogel, cultured overnight in 24-well plates, washed 3 times with PBS, and fixed for 30 ​min in 10% NBF (neutral buffered formalin). After fixation, cells were permeabilized with 0.05% Triton X-100 (Sigma)/PBS for 10 ​min. The cells were incubated in primary antibody (1:500) containing 1% BSA in PBS blocking solution for 1 ​h at 4 ​°C in a humid tray. Afterward, the cells were rinsed twice with PBS, incubated with secondary antibody (1:1000) diluted in PBS for 1 ​h, and stained with DAPI for 2 ​min. Finally, all samples were mounted in aqueous Fluorescent Mounting Medium (Dako, Denmark), and immunofluorescent staining was imaged on an EVOS2 FL Auto 2 live-cell imaging system and analyzed by ImageJ software (NIH, USA).

For morphology observation, cells were cultured for 7 days and fixed and permeabilized as described above. Then, the samples were stained with rhodamine-Phalloidin (F-actin labeling) and DAPI (nuclear staining).

To analyze production of extracellular matrix from the fibroblasts on hydrogels, fibronectin synthesis in the cellular response was examined using immunocytochemistry. Cells were cultured on hydrogel for 7 days and stained with primary antibody (fibronectin, MA5-14737, Thermo Fisher, 1:100) and then with secondary antibody (Alexa-Fluor goat anti-mouse 488, A11029, Thermo Fisher, 1:1000).

### 2.9 Western blot analysis

For western blotting analysis hVFFs (1 ​× ​10^5^ ​cells/well) were cultivated in 6 well plates for 24 ​h hVFF cell lysates were prepared using RIPA buffer (Sigma Aldrich, St Louis, MO, USA) containing protease inhibitor cocktail and PhoSTOP (Roche Molecular Biochemicals, Basel, Switzerland), and the total protein concentration of lysate was estimated using the BCA assay kit (Thermo Scientific, Rockford, MD, USA). Denatured protein samples were separated by SDS-PAGE (10% acrylamide) gel electrophoresis and transferred to nitrocellulose membranes for immunoblotting. Nonspecific binding sites were blocked with 5% skim milk for 1 ​h at room temperature, and the membranes were incubated with primary antibodies overnight at 4 ​°C. After the membranes were washed 4 times for 5 ​min, the blots were incubated with horseradish peroxidase-conjugated secondary antibodies for 1 ​h at room temperature. Secondary antibodies were detected using chemiluminescence reagents (GE Amersham™ ECL; GE Healthcare, IL, USA) using the Amersham™ Imager 680 (GE Healthcare, Chicago, IL, USA). Each hydrogel group were analyzed in triplicate and the data are shown as mean ​± ​standard deviation.

### 2.10 In vivo biocompatibility and biodegradability study

C57BL6 male mice (6 weeks old) were used in biocompatibility experiments (for 3, 7, 14, and 28 days). Mice were housed in a temperature-controlled room and maintained on a 12/12 ​h light/dark cycle. The mice were allowed free access to clean water and standard laboratory pellets. Hair was removed from all animals using depilatory cream (Veet, USA) at 7 weeks of age.

The animals were divided into 3 groups: control (alginate), silk-alginate (S-A) hydrogel, and LTP-treated silk-alginate (n ​= ​3 mice per group). Hydrogels (250 ​μL) were subcutaneously injected into the dorsal skin with a 25G needle. At 3, 7, 14, and 28 days, a mouse from each group was sacrificed to obtain a skin specimen. Visible hydrogel was photographed with a Samsung mobile phone (Galaxy S20, Korea).

Skin specimens were fixed in 10% neutral buffered formalin for 24 ​h, dehydrated, and embedded in paraffin with an automated tissue processor (Spin Tissue Processor STP-120, Thermo Scientific, Germany). Specimens were cut to a thickness of 6 ​μm and mounted on glass slides. Tissue sections were stained with hematoxylin and eosin (H&E) and Masson's trichome for morphological evaluation. After collecting images through a microscope, the area of remaining hydrogel was calculated and compared among groups.

Immunofluorescence analysis showed the expression of Col Ia1 (1:250 dilution, Abcam, USA) and VEGF (1:250, Santa Cruz, USA) in the hydrogel and under the skin of mice after 14 days. Skin sections were deparaffinized and hydrated in a graded series of ethanol. Non-specific binding sites on the hydrated slides were blocked with 2% BSA and 0.02% Triton X-100 in phosphate buffered saline (PBS) for 1 ​h. Thereafter, primary antibodies of ColIa1 and VEGF were applied at 4 ​°C overnight. Secondary antibodies were diluted in PBS (Alexa FluorTM 488 goat anti-rabbit IgG antibody, Invitrogen, USA, diluted 1:200) at room temperature for 1 ​h. Nuclei were counterstained with DAPI. Tissues were mounted in fluorescent mounting medium (Dako, Inc., Switzerland), and images were visualized under an EVOS2 fluorescence microscope.

### 2.11 Vocal fold injury procedures

Eighteen male New Zealand White rabbits with body weights ranging from 2.0 to 2.5 ​kg were used in the experiments. Animal care and experimental methods and procedures were approved by the Animal Experimental Ethics Committee, College of Medicine, Ajou University (No. 2017–0023). Animals were randomly divided into three groups of (1) alginate, (2) silk-alginate (S-A), and (3) LTP-treated silk-alginate (S-A). The animals were anesthetized with tiletamine (5.5 ​mg/kg, Virbac Ltd., France), zolazepam (5.8 ​mg/kg, Virbac Ltd.), and xylazine hydrochloride (5 ​mg/kg, Bayer, Korea) and then placed on the operating table in a supine position. After general anesthesia, the larynx was visualized with a 4 ​mm rigid endoscope (Karl Storz, Germany) inserted transorally. A microscissors was used to injure the right VF including the epithelium, lamina propria, and basal VF muscle. Immediately, 0.2 ​mL of hydrogels was injected into the VF (lateral to injury) with a 25G metal-type spinal needle (Hakko, Japan). Each animal was sacrificed in a CO_2_ chamber after 7 weeks, and all VFs were obtained by laryngectomy.

### 2.12 Examination of vocal fold vibration

To excise the VFs from the obtained larynx, all the glottal structures were removed, and the cartilage was sutured using polygalactin 910, 5-0 suture (Ethicon-coated VICRYL, USA) for VF closure. The VF tissue was fixed in an air tube, vibrated by expiratory airflow, and recorded with a high-speed digital imaging system (NX4-S2, Integrated Design Tools, Tallahassee, FL, USA) at 5000 frames per second (fps). All tissues experiments were performed in triplicate, and kymographs were automatically generated using Metamorph® NX imaging software. Kymographs were used to calculate the amplitude of mucosal oscillations on both sides of the VFs as the ratio of open to closed phases.

### 2.13 Histopathological and immunofluorescence examination

After kymography, the VF tissues were harvested for histological analysis. The specimens were embedded in paraffin as block specimens and then sectioned with a thickness of 6 ​μm using a microtome. The specimens were stained with hematoxylin and eosin (H&E), Masson's trichrome (MT), and Alcian blue staining and observed under an EVOS2 microscope.

Immunofluorescence analysis for Col Ia1 (1:250, Abcam, USA) and fibronectin (1:250, Invitrogen, USA) was performed to evaluate regeneration. Samples were blocked with 2% BSA and primary antibody in a refrigerator overnight. Secondary antibody was incubated at room temperature for 1 ​h (1:200, Alexa FluorTM 488 goat anti-rabbit IgG antibody, Invitrogen, USA.) and nuclei were stained with DAPI. Imaging was performed with an EVOS2 fluorescence microscope.

### 2.14 Statistical analysis

All analyses were performed using the GraphPad Prism 5 package (GraphPad Software Inc., USA). Data are expressed as mean ​± ​standard deviation (SD). Tukey's multiple comparisons test followed one-way Analysis of Variance (ANOVA) test to verify the results. A value of ∗*p* ​< ​0.05 was considered statistically significant.

## 3 Results

### 3.1 Liquid-type plasma affected the mechanical properties of silk-alginate hydrogel dose-dependently

**Fig. 1:**
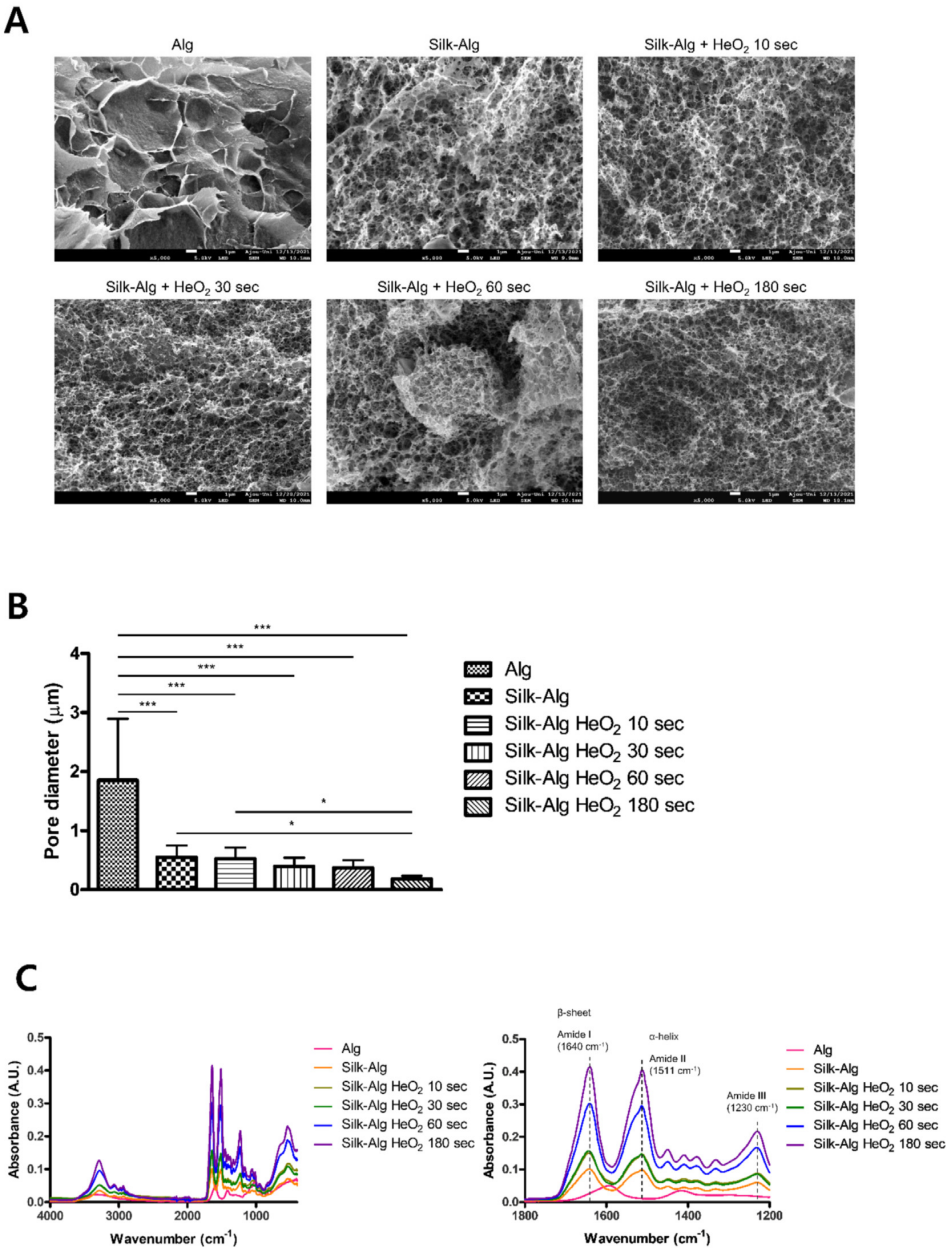

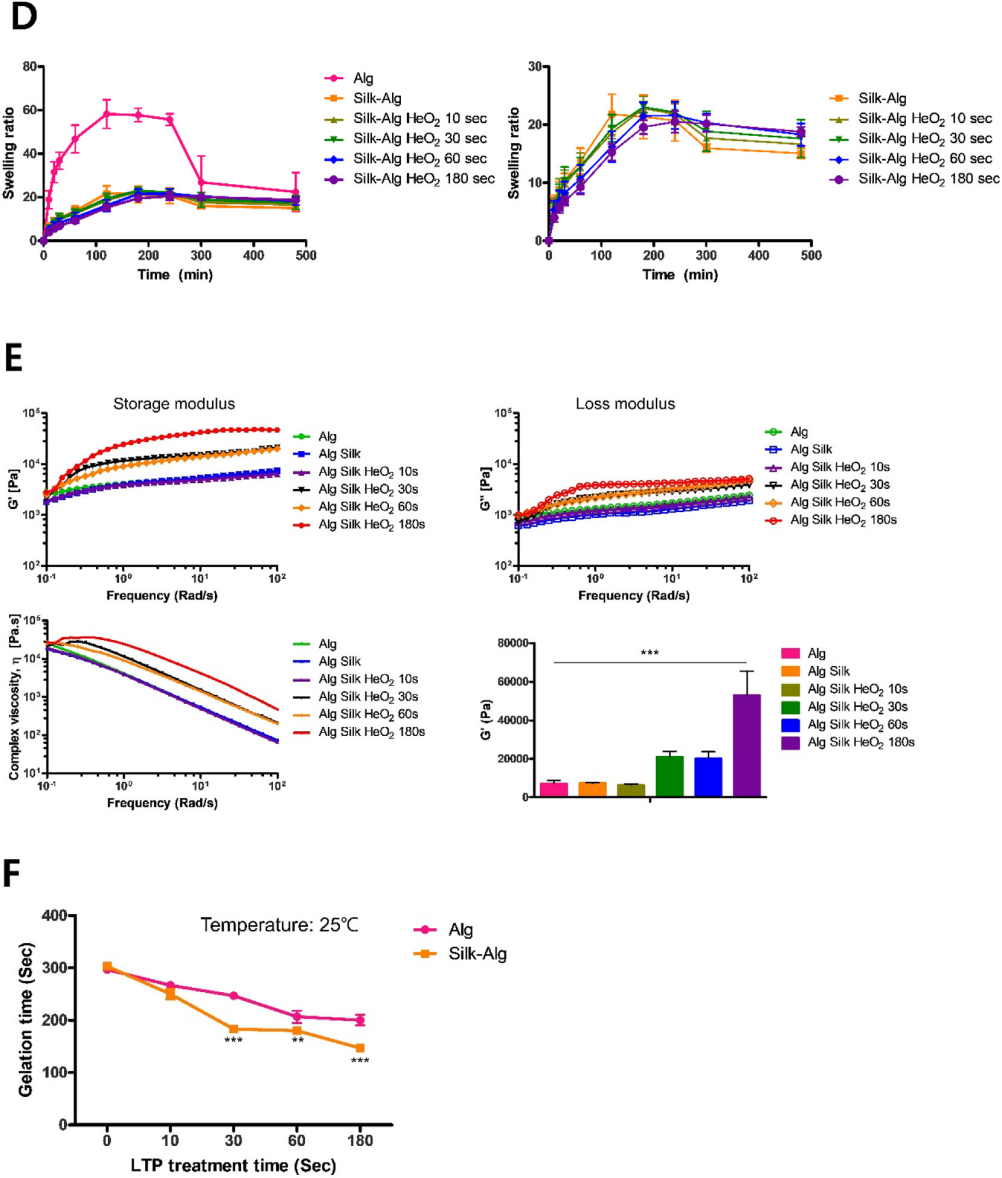
Alterations of microstructures and mechanical properties of alginate hydrogel after adding silk and liquid-type plasma (LTP).

The surface morphologies of the hydrogel were observed by SEM. On the surface of the S-A hydrogel, a silk membrane lamellar microstructure that was not seen in the alginate hydrogel was observed, and a larger area of the silk membrane lamellar microstructure was observed as LTP treatment time increased (Fig. 1A). In addition, pore size decreased significantly in the order of alginate, S-A, and LTP-treated S-A hydrogel (Fig. 1B). When a secondary structure was formed by crosslinking of silk, a peak was observed for the amide I (1700–1600 ​cm^−1^), amide II (1600–1500 ​cm^−1^), and amide III (1350–1200 ​cm^−1^) regions, which are characteristic wavelength bands in FTIR. In addition, the alginate hydrogel showed a peak in a region not related to the secondary structure of silk. The LTP-treated S-A hydrogel and S-A hydrogel showed peaks in the amide I, II, and III regions, and the LTP-treated S-A hydrogel showed higher intensity than the S-A hydrogel regardless of LTP treatment time (Fig. 1C). Next, the swelling ratio test was performed to investigate the mechanical properties of the hydrogel, and the results are shown in Fig. 1D. Before swelling ratio test, the weights of freeze-dried hydrogels were measured (Figure S1). Unlike other hydrogels, alginate hydrogel showed a rapid early increase of swelling ratio. After reaching the peak, when the alginate hydrogel was removed from the water to measure the weight (wet), the surface became brittle and fell off, which resulted in a decrease in the swelling ratio. The LTP-treated S-A hydrogels reached the peak swelling ratio later than did the S-A hydrogel and only the S-A hydrogels treated with LTP for 60 ​s and for 180 ​s maintained the ratio after the peak, whereas the swelling ratio of S-A hydrogel and other LTP-treated S-A hydrogels decreased soon after the peak. The appearance of surface peeling decreased as the LTP treatment time increased. As crosslinking increases, the decrease in the swelling ratio of the hydrogel is reduced, indicating that the crosslinking inside the S-A hydrogel increases with LTP treatment time [29,30]. Rheology analysis was performed to investigate the viscoelastic mechanical properties of hydrogel. The S-A hydrogel treated with LTP for 10 ​s exhibited storage (G′) modulus, loss (G″) modulus, and complex viscosities similar to those of alginate hydrogels and S-A hydrogels, but as LTP treatment time increased beyond 30 ​s, the values of the 3 indicators increased (Fig. 1E). The maximum G′ of the S-A hydrogel treated with LTP for 180 ​s was significantly the highest. A case with a larger G′ value than G″ value can be regarded as a solid-like material [31], and all hydrogels showed solid-like properties (Figure S2). The gelation time was measured at 25 ​°C (Fig. 1F). We attempted to measure gelation time of hydrogel solution injected through n that occurs when the hydrogel solution is injected through the 25 ​G syringe. However, gelation occurred immediately upon exit from the syringe (Video 1). The gelation time of alginate and S-A hydrogel were 300 ​s without LTP treatment. LTP treatment reduced the gelation times of all hydrogel. Moreover, as the LTP treatment time increased, the gelation times of LTP treated S-A hydrogels significantly decreased compared to those of LTP-treated alginate hydrogel. Based on these results, LTP induced crosslinking of silk in the S-A hydrogel and changed its mechanical properties.

(A) Scanning electron microscope (SEM) images of freeze-dried alginate hydrogel, S-A hydrogel, and LTP-treated S-A hydrogel with different HeO_2_ treatment times of 10 ​s, 30 ​s, 60 ​s, and 180 ​s (magnification 5000 ​× ​, scale bar of 1 ​μm). (B) The pore size of the hydrogels under SEM significantly decreased in the order of alginate, S-A, and LTP-treated S-A hydrogel. The data were analyzed using a *t*-test (∗, *P ​< ​0.05*, ∗∗, *P ​< ​0.01*, ∗∗∗, *P ​< ​0.001*). (C) Secondary structural analysis by Fourier-transform infrared (FTIR). Raw average FTIR spectra are presented in the graph on the left. The three amide band regions including the amide I representing β-sheet were not revealed in alginate hydrogel but were observed in S-A hydrogel. As shown in the graphs on the right, the peak intensity of the three amide band regions increased as LTP treatment time increased. (D) S-A hydrogels had lower swelling ratio than alginate hydrogel. The left graph shows that the time to reach the peak swelling ratio was delayed as LTP treatment time was increased, and reduction of swelling ratio after the peak was decreased. (E) Rheology test showed increased storage modulus (G′), loss modulus (G″), and complex viscosity of LTP-treated S-A hydrogels as LTP treatment time increased. The data were analyzed using a *t*-test (∗, *P ​< ​0.05*, ∗∗, *P ​< ​0.01*, ∗∗∗, *P ​< ​0.001*). (F) Gelation time of each hydrogels.

### 3.2 Controlled release of the plasma effectors of reactive nitrogen species from silk-alginate hydrogel

**Fig. 2:**
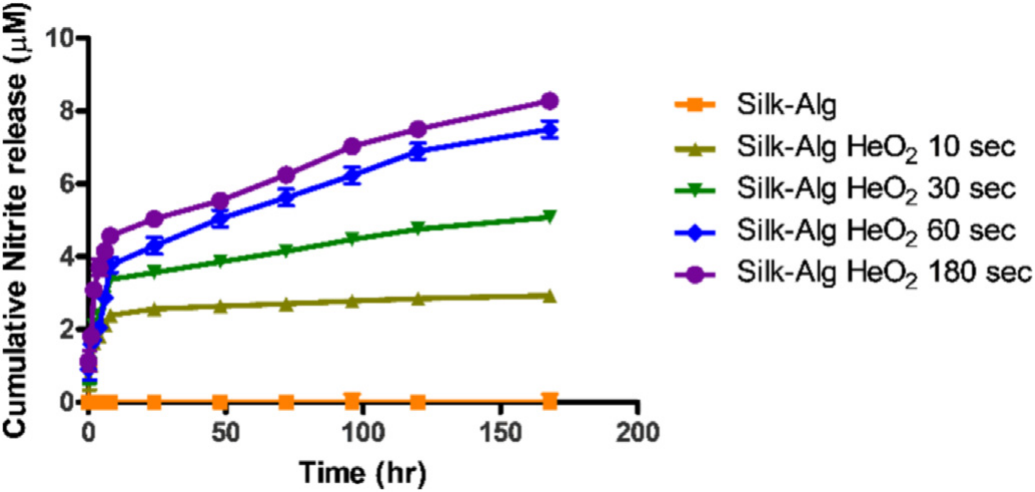
Controlled release of nitrite from hydrogels.

One of the advantages of LTP is that it releases RONS, which are helpful for wound healing. Reactive nitrogen species, one of the main effectors of LTP, are more highly involved in wound healing than are reactive oxygen species, so the release of reactive nitrogen species was measured in the form of nitrite. Measurement of cumulative nitrite release for 7 days showed that nitrite was continuously released from the LTP-treated S-A hydrogel, and more nitrite was released at LTP treatment time of 60 ​s–180 ​s compared to that of 10 ​s–30 ​s (Fig. 2). This suggests that LTP-treated S-A hydrogel could contribute to wound healing through controlled release of reactive nitrogen species. Considering the collective results of mechanical properties and nitrite release, subsequent experiments were conducted with LTP treatment time up to 60 ​s.

Nitrite was consistently released from LTP-treated S-A hydrogel, and the total amount of released nitrite increased as LTP treatment time increased.

### 3.3 The liquid-type plasma-treated silk-alginate hydrogel increased extracellular matrix deposition and migration of vocal fold fibroblast

**Fig. 3:**
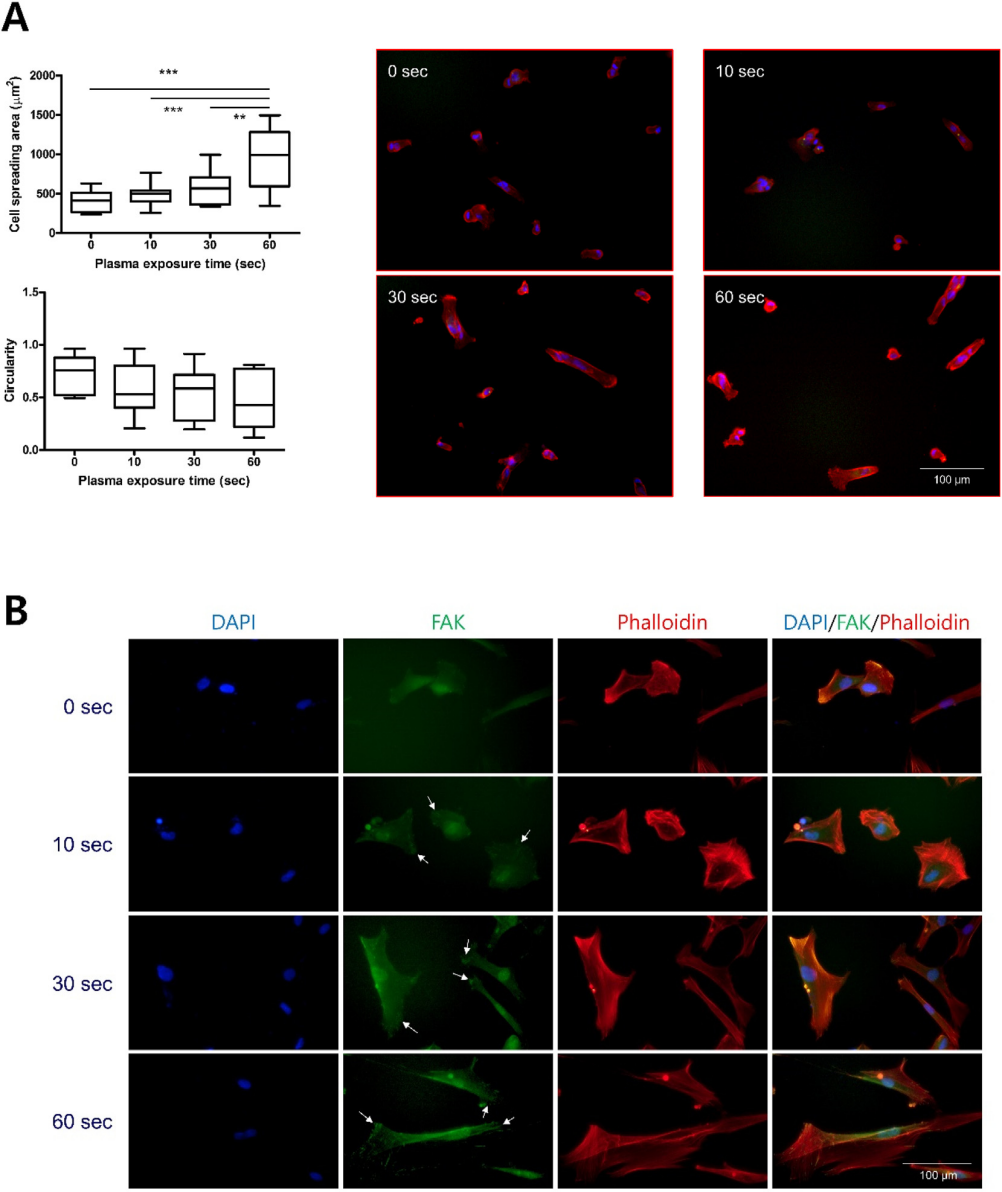

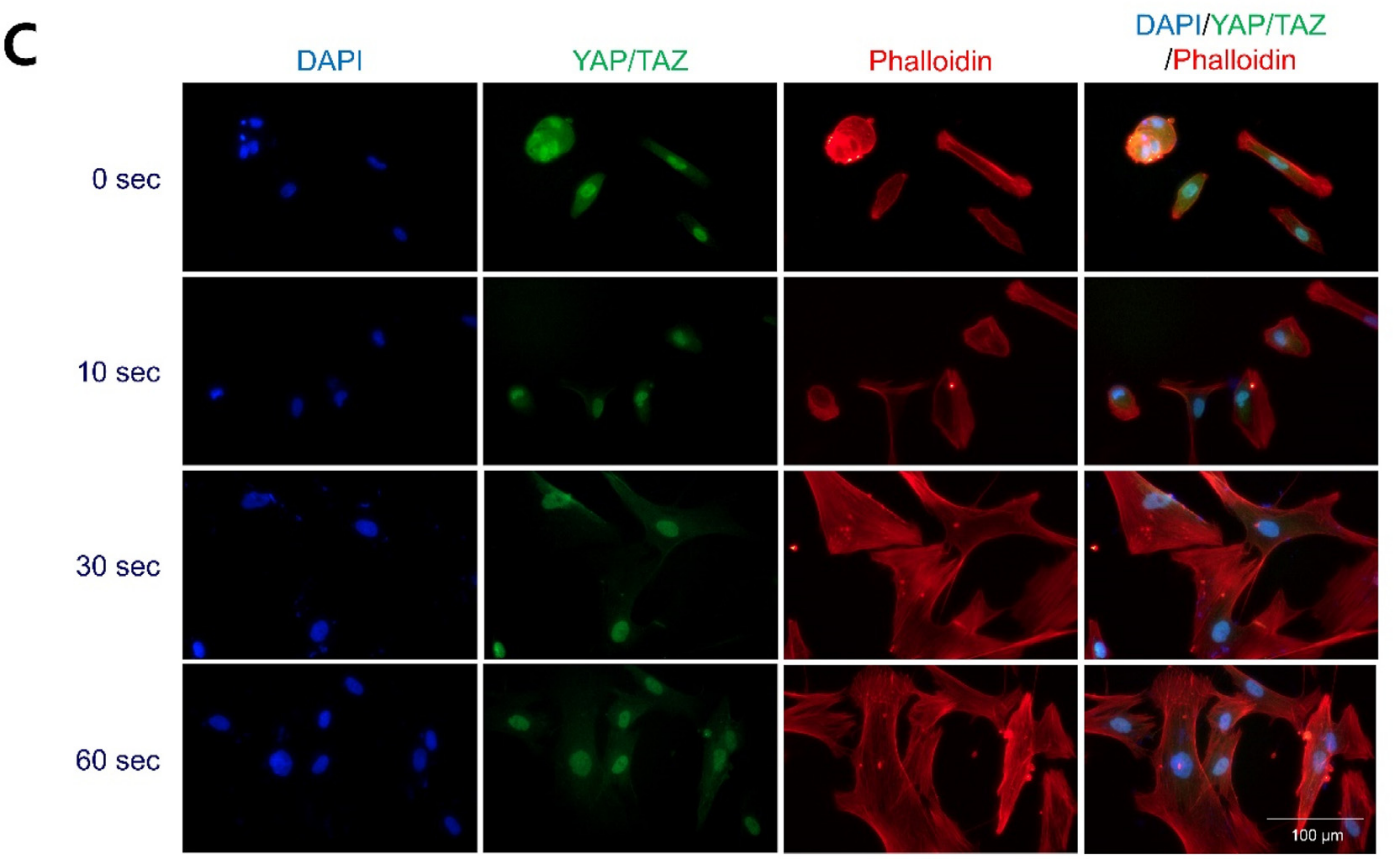

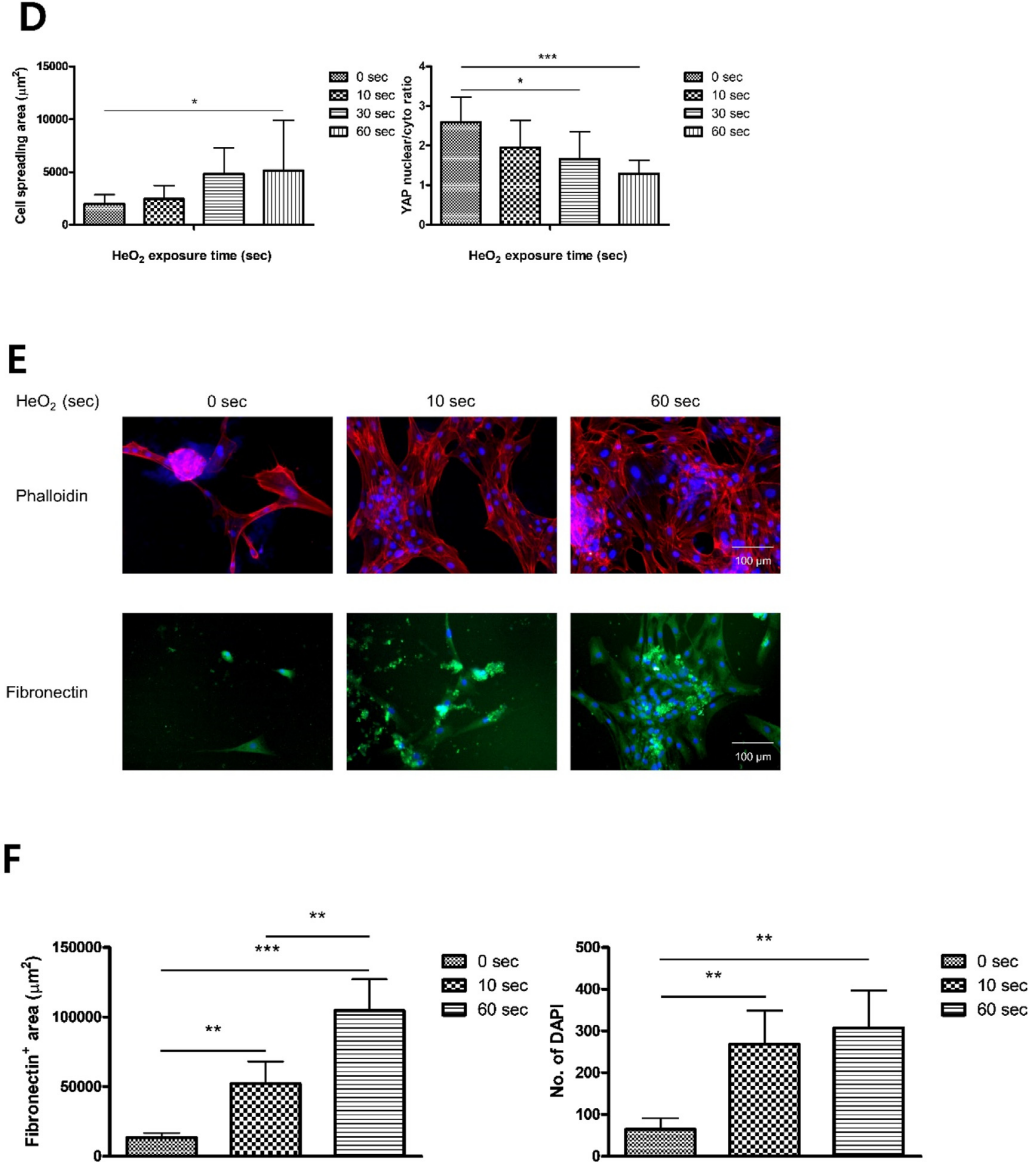

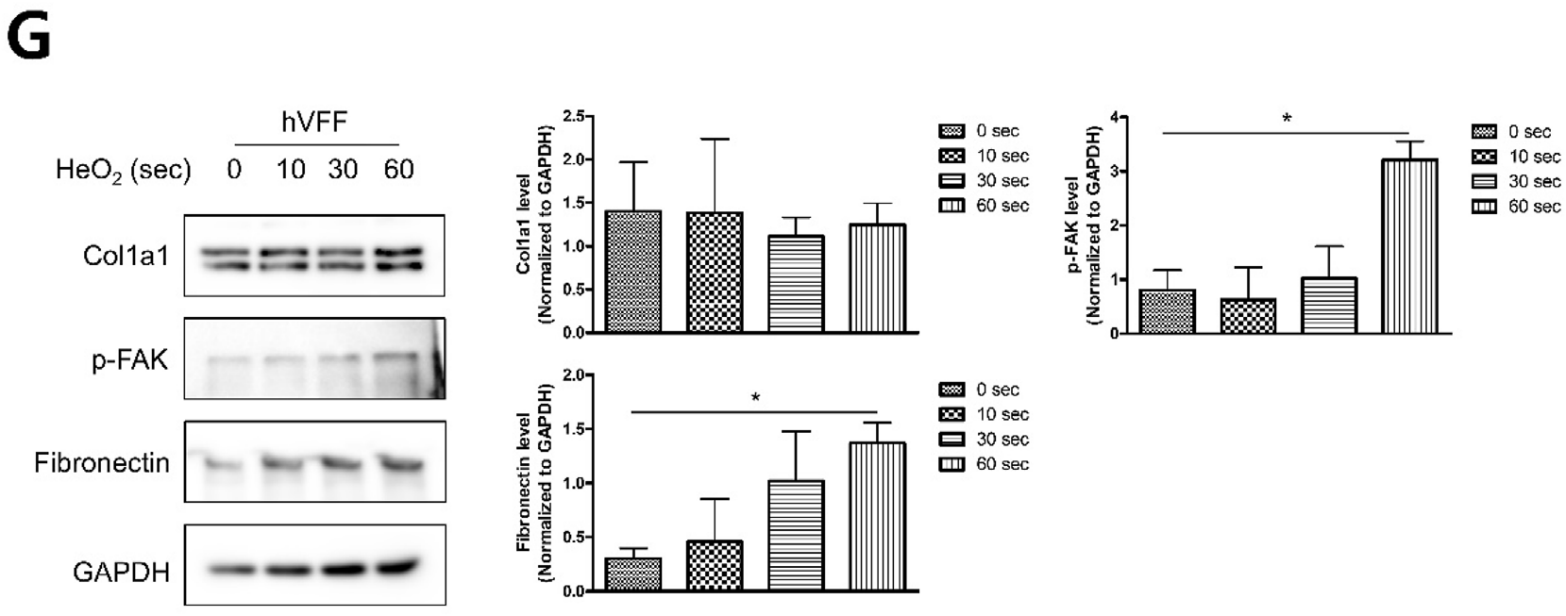
Effects of liquid-type plasma (LTP) treatment on the mobility and extracellular matrix (ECM) synthesis of human vocal fold fibroblast (hVFF).

To investigate the effect of LTP-treated S-A hydrogel on hVFF, cell adhesion area and circularity of hVFF were measured. When the LTP treatment time was 60 ​s, the cell adhesion area was significantly increased, and the circularity was lowest of all tested times (Fig. 3A). In FAK and YAP/TAZ staining, the mobility of hVFF increased as LTP treatment time increased (Fig. 3B and C), with a significant difference according to exposure time (Fig. 3D). Fibronectin is an important component of ECM in wound healing [32]. *In vitro* ECM deposition was analyzed by fibronectin immunofluorescence staining of hVFF. As LTP treatment time increased, the expression level of fibronectin on the surface of S-A hydrogel significantly increased (Fig. 3E), as confirmed by quantitative analysis of fibronectin area and number of DAPI-stained cells (Fig. 3F). The expression of p-FAK, fibronectin, and ColA1 was quantitatively analyzed by ELISA and western blot. As shown in immunofluorescence, the expression of fibronectin and p-FAK significantly increased, and the expression of Cola1 showed a tendency to increase as LTP treatment time increased (Fig. 3G). Based on the experimental results, LTP can increase fibroblast mobility and ECM synthesis. An LTP treatment time of 60 ​s did not produce cytotoxicity and was used for all experiments.

(A) hVFFs were incubated on LTP-treated S-A hydrogel for 7 days. In immunocytochemistry with Phalloidin, the cell spreading area significantly increased and circularity decreased as LTP treatment time increased. The data were analyzed using a *t*-test. Scale bar: 100 ​μm. (B) Immunocytochemisty with FAK revealed increased expansion in hVFF area as LTP treatment time increased. Blue: DAPI, green: FAK, red: Phalloidin. Scale bar: 100 ​μm. (C) Immunocytochemisty with YAP/TAZ revealed increased YAP in the nuclei as LTP treatment time increased. Blue: DAPI, green: YAP/TAZ, red: Phalloidin. Scale bar: 100 ​μm. (D) Statistical analysis showed significantly increased expression of FAK and YAP/TAZ. The data were analyzed using a *t*-test (∗, *P ​< ​0.05*, ∗∗, *P ​< ​0.01*, ∗∗∗, *P ​< ​0.001*). (E) Immunofluorescence of DAPI and fibronectin assay after LTP treatment showed increased ECM synthesis and proliferation of hVFF. Blue: DAPI, green: fibronectin, red: Phalloidin. (F) Statistical analysis showed significant increases in the expression of fibronectin and number of DAPI-stained cells. The data were analyzed using a *t*-test (∗, *P ​< ​0.05*, ∗∗, *P ​< ​0.01*, ∗∗∗, *P ​< ​0.001*). Scale bar: 100 ​μm. (G) The results of western blot showed significant increase of fibronectin and p-FAK of hVFF with LTP treatment time.

### 3.4 The biocompatibility and biodegradation of the liquid-type plasma-treated silk-alginate hydrogel in mice

**Fig. 4:**
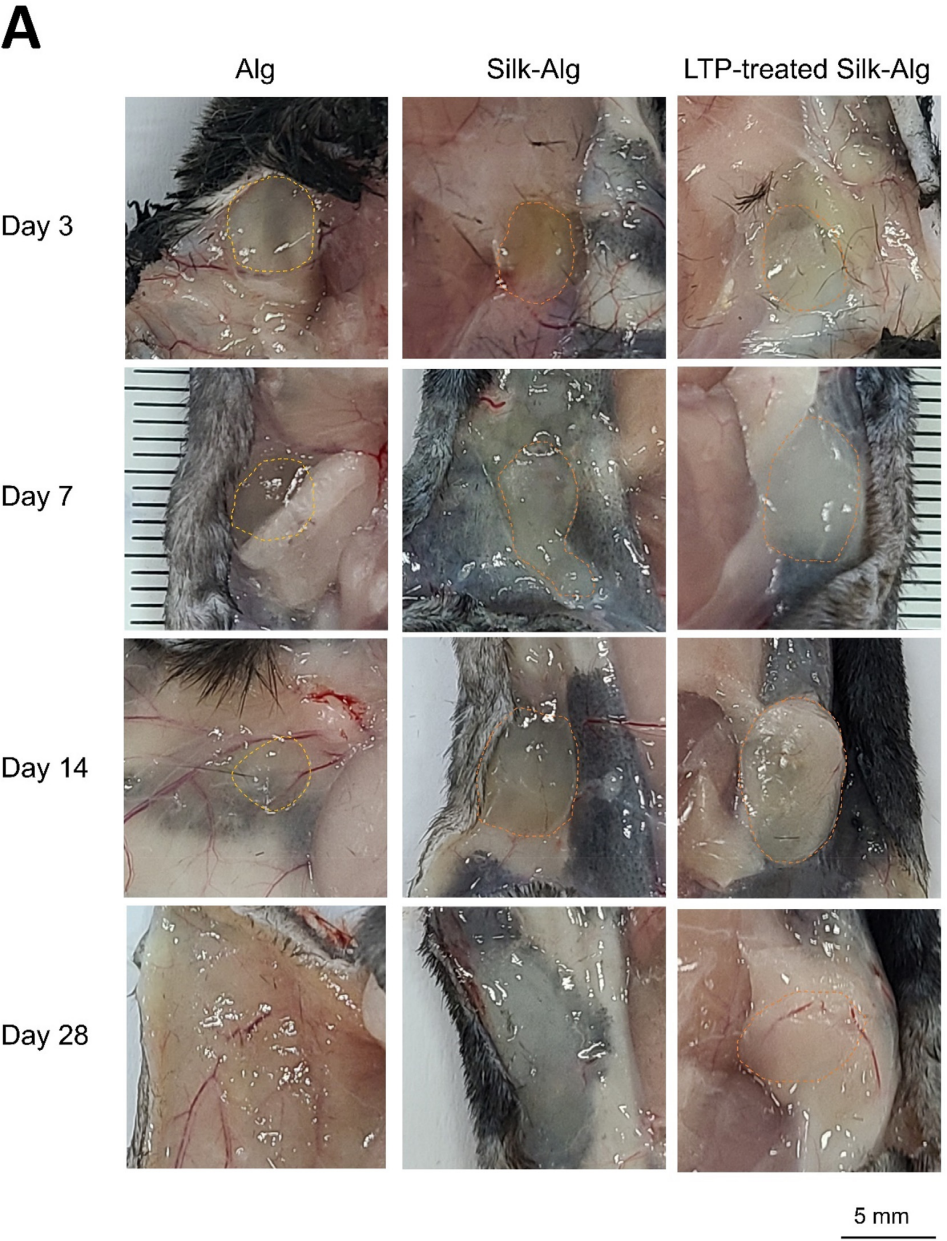

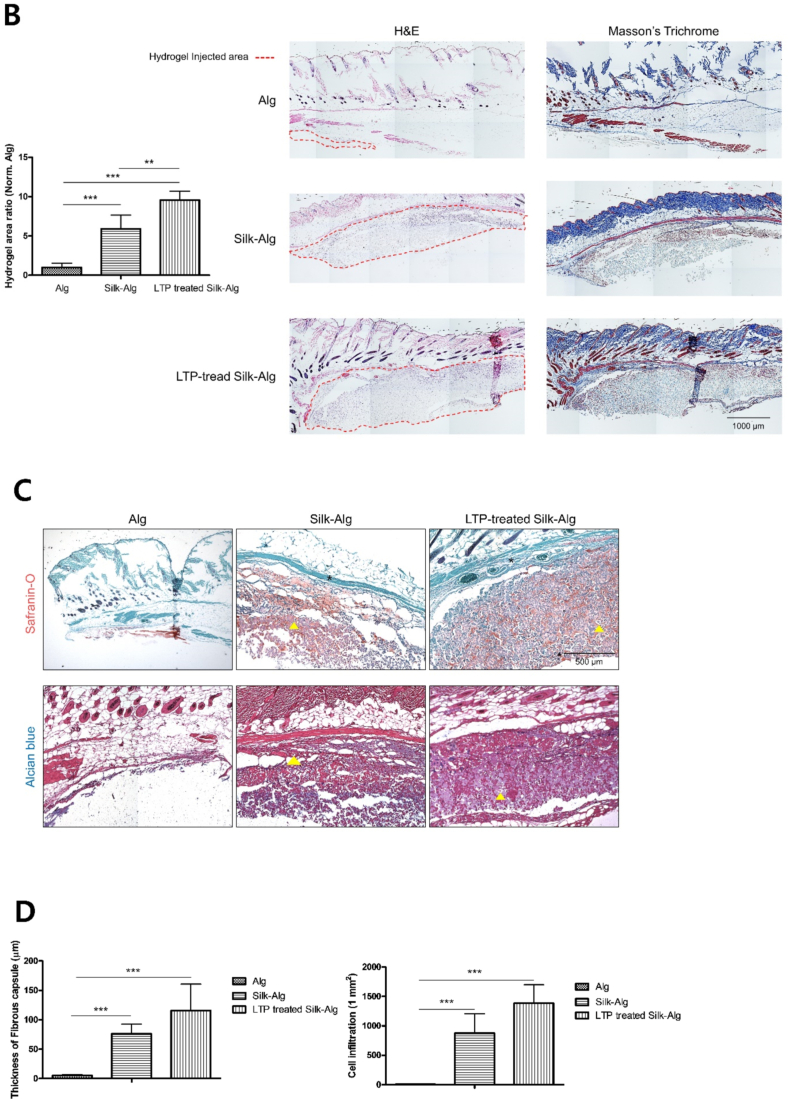

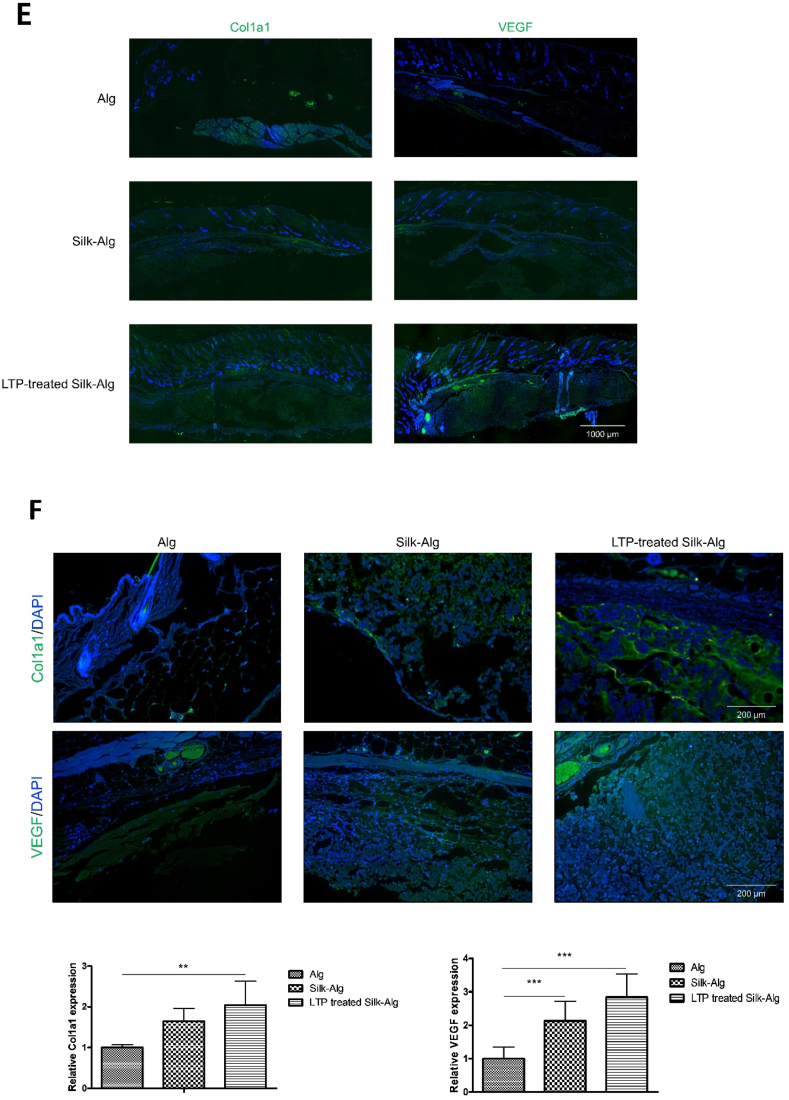
The biocompatibility and biodegradability of alginate, silk-alginate (S–A), and liquid-type plasma (LTP)-treated silk-alginate (S–A) hydrogel in mice.

Hydrogel was injected subcutaneously in mice to evaluate biocompatibility and biodegradation. Little alginate hydrogel remained on Day 14; while most of the S-A hydrogel was absorbed by Day 28, LTP-treated S-A hydrogel was maintained (Fig. 4A). Histological analysis on Day 14 revealed absorption of most of the alginate hydrogel and significant absorption of the S-A hydrogel compared to LTP-treated S-A hydrogel (Fig. 4B). When Safrain-O and Alcian blue staining was performed, significantly thicker surrounding fibrous capsules and higher cell infiltration were observed in the LTP-treated S-A hydrogel compared to other hydrogels (Fig. 4C and D). Immunofluorescent staining of Col1A1 and VEGF was performed to examine ECM synthesis and angiogenesis. The expression of Col1A1 and VEGF was observed in a wider area in LTP-treated S-A hydrogel compared to other hydrogels (Fig. 4E), and the expression level of Col1A1 was significantly higher in LTP-treated S-A hydrogel (Fig. 4F).

(A) At Days 3, 7, 14, and 28 after subcutaneous injection of hydrogels. The remaining hydrogel is indicated by a dash line. Scale bar: 5 ​mm ​(B) Remaining hydrogel at Day 14 was seen under H&E and Masson's Trichrome staining. The ratios of remaining hydrogel to alginate hydrogel were analyzed using a *t*-test. Scale bar: 1000 ​μm. (C) Under Safrain-O and Alcian blue staining, surrounding fibrous capsules (∗) and cell infiltration (yellow triangle) increased most highly in the LTP-treated S-A hydrogel, followed by S-A hydrogel and alginate hydrogel. Scale bar: 500 ​μm. (D) Statistical analysis showed significantly highest thickness of fibrous capsules and cell infiltration in the LTP-treated S-A hydrogel. The data were analyzed using a *t*-test. (∗, *P ​< ​0.05*, ∗∗, *P ​< ​0.01*, ∗∗∗, *P ​< ​0.001*). Scale bar: 1000 ​μm. (E) Immunofluorescent staining of Col1A1 and VEGF. Scale bar: 1000 ​μm. (F) The expression level of Col1A1 and VEGF were significantly higher in LTP-treated S-A hydrogel. The data were analyzed using a *t*-test (∗, *P ​< ​0.05*, ∗∗, *P ​< ​0.01*, ∗∗∗, *P ​< ​0.001*).

### 3.5 Regenerative effect of the liquid-type plasma-treated silk-alginate hydrogel in vocal fold injury *in vivo*

**Fig. 5:**
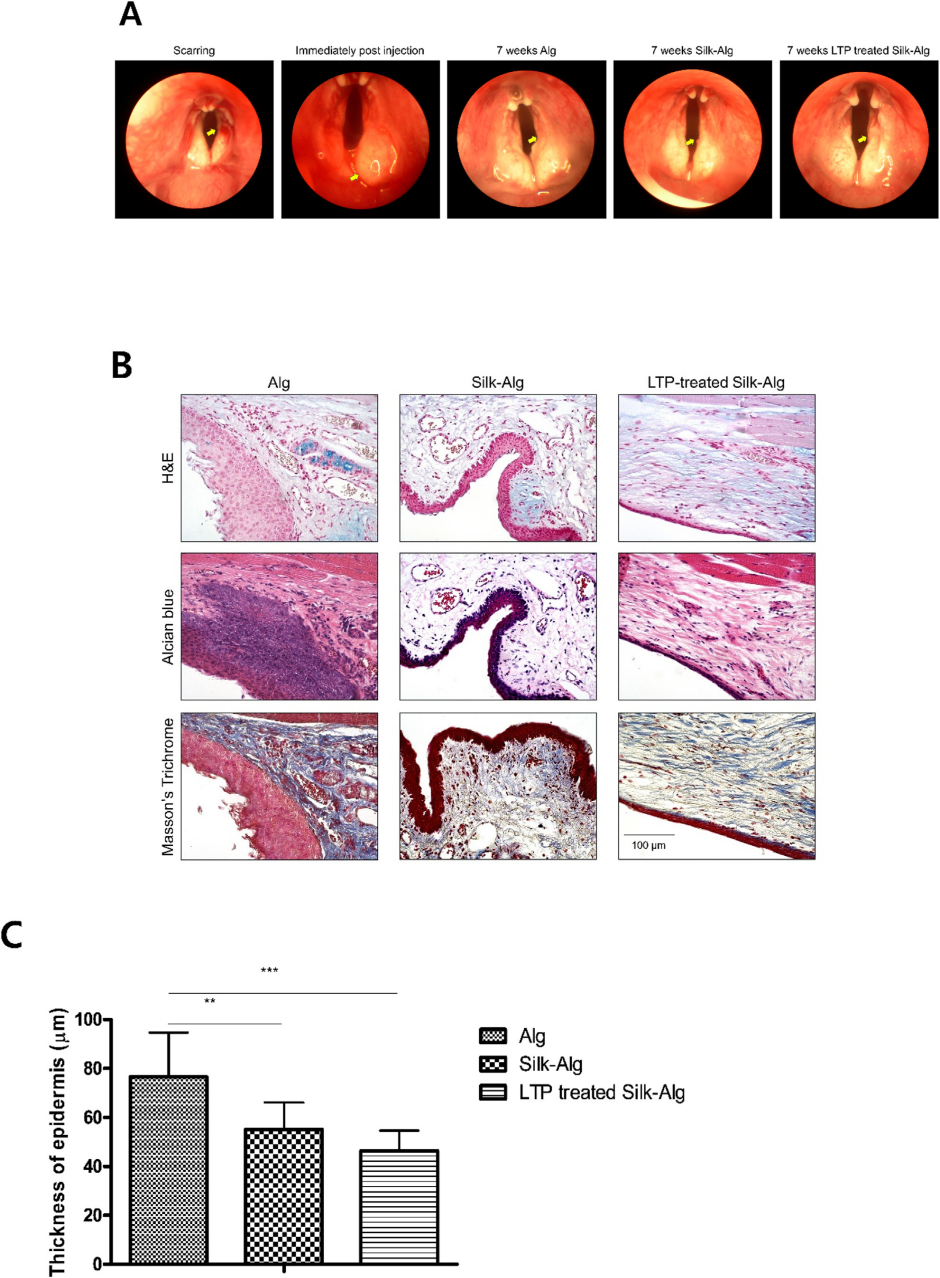

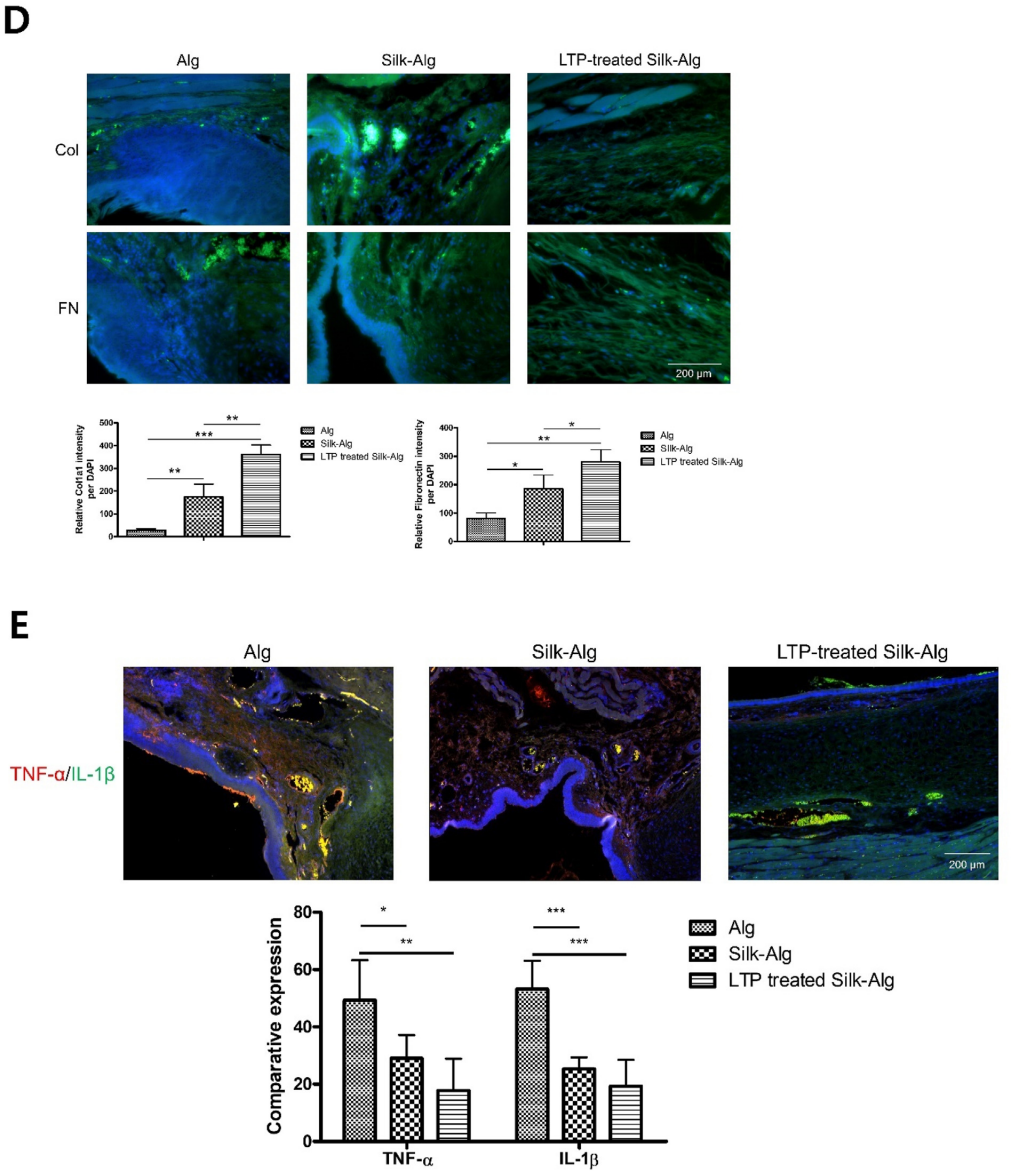

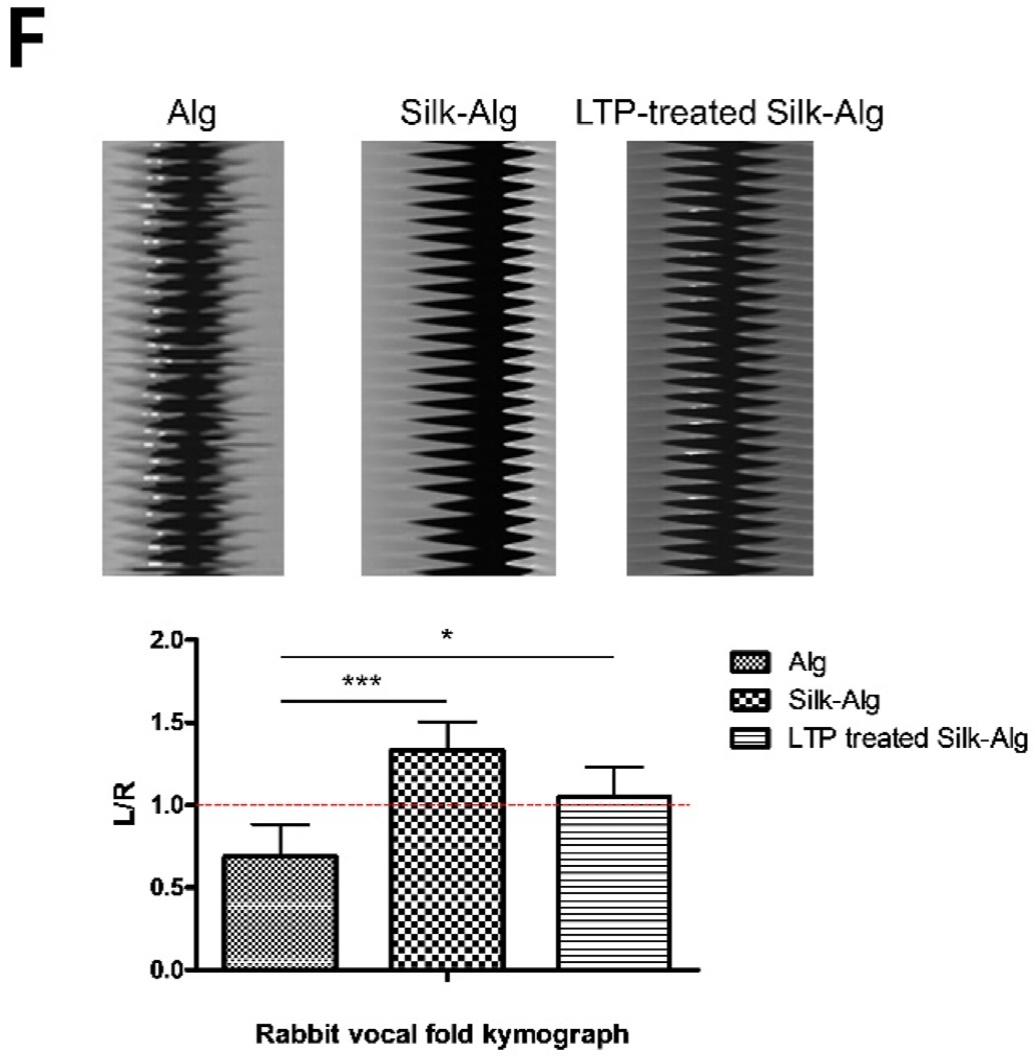
Wound healing effects of alginate, silk-alginate (S–A), and liquid-type plasma (LTP)-treated silk-alginate (S–A) hydrogel in a vocal fold injury model.

After creating a full-thickness wound on the right VF in rabbit with microscissors, 200 ​μL hydrogels were injected into the lateral side of the wound through a 25-gauge needle. When the VFs were observed through an endoscope at 7 weeks, contractions due to scarring were seen in the group injected with alginate hydrogel, but none were observed in the group injected with S-A hydrogel or LTP-treated S-A hydrogel (Fig. 5A). The regenerative effect was evaluated through histologic analysis at 7 weeks after injection of hydrogel. To investigate scar formation, the thickness of epidermis was measured on H&E staining [33]. The epithelium was significantly thicker in the order of alginate hydrogel, S-A hydrogel, and LTP-treated S-A hydrogel. On Alcian Blue and Masson's trichrome staining, more connective tissue was formed in LTP-treated S-A hydrogel than in the other hydrogels (Fig. 5B and C). Lamina propria are important structures for vibration of VFs and are rich in collagen type I [34]. On immunofluorescent staining of Col1A1 and fibronectin, their expression was significantly increased in LTP-treated S-A hydrogel (Fig. 5D). Moreover, even distribution of Col1A1 compared to S-A hydrogel seemed to indicate reduced scar formation [35]. Interleukin-1β (IL-1β) and tumor necrosis factor-α (TNF-α) were measured to evaluate inflammatory response (Fig. 5E). The expression of TNF-α/IL-1β was significantly lower in LTP-treated S-A hydrogel. Videokymographic analysis was performed to determine the degree of functional recovery of the regenerated VF mucosa. The amplitudes of the VF mucosal waves in alginate and S-A hydrogel were different from those of the normal VF, but those in LTP-treated S-A hydrogel were similar to those of normal VF (Fig. 5F).

(A) Endoscopic images at scarring, immediately post injection, and 7 weeks after injection. (B) H&E, Alcian Blue, and MT staining was performed. Scale bar: 100 ​μm. (C) The thickness of the skin was significantly thinned in LTP-treated S-A hydrogel. The data were analyzed using a *t*-test (∗, *P ​< ​0.05*, ∗∗, *P ​< ​0.01*, ∗∗∗, *P ​< ​0.001*). (D) The fluorescence intensities of Col1a1 and fibronectin significantly increased in the LTP-treated S-A hydrogel. The data were analyzed using a *t*-test. Scale bar: 200 ​μm. (E) The immunofluorescence of tumor necrosis factor-alpha/interleukin-1beta showed reduced inflammatory response in the LTP-treated S-A hydrogel. Scale bar: 200 ​μm. The data were analyzed using a *t*-test (∗, *P ​< ​0.05*, ∗∗, *P ​< ​0.01*, ∗∗∗, *P ​< ​0.001*). (F) Representative kymographic images are presented for each group. The amplitude ratios of mucosal waves in treated VFs relative to normal contralateral VFs were close to 1 in the LTP-treated S-A hydrogel, significantly different from the other hydrogels. The data were analyzed using a *t*-test (∗, *P ​< ​0.05*, ∗∗, *P ​< ​0.01*, ∗∗∗, *P ​< ​0.001*).

## 4 Discussion

Hydrogel consists of a three-dimensional crosslinked hydrophilic polymer network and can retain a large amount of water. Due to the water storage property, hydrogel presents biocompatibility and can encapsulate substances such as cells, proteins, and drugs for various medical purposes [36]. Moreover, tunable mechanical properties including stiffness and viscosity of hydrogel have made it possible to load them with injectable agents, as has gained interest because of their advantages of minimal invasiveness. Injectable hydrogel involves administration of sol or pre-gel at a target site for gradual “sol-gel” transition, known as *in situ* gelation, inside the living body. *In situ* gelation is essential for injectable hydrogels and should occur timely in easily achieved conditions without harmful stimulus [37]. Various physical and chemical crosslinking methods have been applied for *in situ* gelation, including ionic crosslinking, photo-crosslinking, stimuli sensitive crosslinking, and enzymatic crosslinking [36,37]. Since the ideal injectable hydrogel requires injectability, timely gelation after injection, biocompatibility, and biodegradability, it is not easy to design an appropriate crosslinking method [3]. Herein, we applied an LTP technique for *in situ* gelation of S-A injectable hydrogel for VF regeneration.

In this investigation, we produced an S-A composite gel with LTP and then examined the changes of mechanical properties and regenerative potential compared to those of alginate-only or S-A composite gel. The advantages of current hydrogel compared to previously reported hydrogels are fast gelation within several seconds with non-cytotoxic *in situ* crosslinking and regenerative potential without need for bioactive materials such as stem cell and growth factor. *In situ* crosslinking for injectable hydrogel applied to superficial tissue is induced by photo-crosslinking, stimuli sensitive crosslinking. These methods are difficult to apply in vocal fold injury due to deep location and high mobility of vocal fold. *In situ* crosslinking via additives is realistic, but there is no effective additive without cytotoxicity. Fast gelation is important considering operation time and encapsulation. Injection of hydrogel into vocal fold is a delicate procedure that requires real-time voice feedback to determine the proper amount of injection. Moreover, if injectable hydrogel contains bioactive materials, gelation must occur before the materials has spread to the surrounding tissues to enable continuous secretion of materials. To improve the regenerative power of hydrogel in vocal fold injury, several studies have added bioactive materials including stem cell and growth factor [38]. Compared with the proposed hydrogel, those need an additional step to add those materials that pose a potential carcinogenic risk. In summary, our method allows rapid gelation and regenerative potential of hydrogels using a simple and safe crosslinking method.

Silk has been used clinically for regeneration of various human tissues, such as peripheral nerve, cornea, skin, ligament, or heart valve [39]. Because hydrogel can be flexible and match the physical and mechanical properties of various biological tissues, many studies using silk-based injectable agents have been conducted [8]. There are several limitations in using the existing conventional crosslinking methods to make an injectable hydrogel. The physical crosslinking methods require a long gelation time; ultrasonication of a silk solution requires tens of hours to become hydrogel [10]. The chemical crosslinking methods have the advantage of a shorter gelation time than the physical crosslinking methods, but they have the disadvantage that cytotoxic agents must be removed before usage. Moreover, one of the requirements of injectable hydrogel is that gelation occurs after injection, prohibiting use of the previously investigated two crosslinking methods for clinical application [7,10]. In order to overcome these limitations, various additives have been used for faster crosslinking without cytotoxicity [1]. Conversely, hydrogel created with natural polymer additives would experience faster crosslinking with relatively simple preparation, although the process remains difficult to precisely control temporally and mechanically [8]. Among the materials used as additives, alginate is a well-known biomaterial widely used in various fields such as drug delivery, wound dressing, and bone regeneration [40]. When silk is mixed with alginate in composite hydrogel, crosslinking occurs between silk and natural polymers through hydrogen bonding. However, this interaction is relatively weak and can lead to uncontrollable degradation, which often requires additional crosslinking [41]. Alginate can rapidly form stable hydrogels with divalent ions such as magnesium, calcium, strontium, and barium [42], and research on biomedical application of S-A composite hydrogel is being conducted [7]. Calcium is mainly used to produce alginate hydrogel as it requires weeks to achieve sufficient crosslinking with silk [43]. If the crosslinking of silk is needed in S-A composite hydrogel, a rather complicated process is required [7,10]. In this study, we induced simple *in situ* crosslinking of silk by mixing LTP, which is non-toxic to living bodies, in the injected S-A hydrogel. Accelerated gelation was observed after LTP treatment due to increased crosslinking of the silk, which was confirmed by smaller pore size on SEM; higher intensity of amide regions on FTIR; and lower swelling ratio, and higher G′, G″, and complex viscosity in a rheology test compared to the hydrogel without LTP. Moreover, small pore size (<1 ​μm) promoted the migration and adhesion of fibroblasts. Cell adhesion to the surfaces of scaffold is important for initiation of cell proliferation and differentiation [44]. Lee et al. reported that osteoblasts showed a spindle shape in small micropore sizes of scaffold (0.2–1.0 ​μm of diameter) and a more spherical cell shape with fewer fillopodia and lamellipodia in large micropore sizes (3.0–8.0 ​μm of diameter) [45]. Wang et al. also demonstrated the promotion of fibroblast adhesion in hydrogels with smaller pore sizes [46].

Plasma-liquid systems have drawn attention in regenerative medicine due to their high potential in material processing and nanoscience, environmental remediation, and tissue regenerative processes [[47], [48], [49]]. Because it can be applied as a liquid, LTP can be easily mixed with injectable hydrogels to modify physical and chemical properties of materials and function biologically in a living body. In biomaterial processing using LTPs, attention is focused on reactions occurring at the LTP-material interface [49]. Several studies have reported that plasma treatment produces crosslinking of protein and polysaccharide [22,50]. Among the RONS formed by plasma, free radical including ^•^OH, H_2_O_2,_ NO_x_, and O atom are presumed to change the structure of the protein through C–H, C–O, and C–N bonds leading to amide formation [21]. Takai et al. studied the plasma induced chemical modification of amino acids and reported that hydroxylation or nitration occurred only in aromatic ring of tyrosine among the representative amino acids (glycine, alanine, serine) constituting silk [51,52]. One of possible mechanism of LTP induced crosslinking is phenolic polymers which is similar to enzymatic crosslinking using H_2_O_2_ [53]. An Indeed, material functionalization can be attributed to the several physical and chemical reactions occurring in the plasma-liquid interface, including oxidation, reduction, or sputtering [49]. Further study should clarify the various mechanisms through which plasma contributes to the bio-function of silk.

In this investigation, LTP-treated S-A hydrogel continuously released the representative effector of nitrite for up to 7 days, and injection of LTP-treated S-A hydrogel promoted wound healing in acute VF injury in rabbit. The RONS generated during plasma treatment are being studied as important substances for wound healing and tissue regeneration [54]. Since nitric oxide (NO), a reactive nitrogen species, plays an important role in wound healing, including inflammatory response, cell proliferation, collagen formation, antimicrobial action, and angiogenesis, research on nitric oxide-releasing biomaterials is active [55]. NO is highly reactive and reacts with other reactive species to form ONOO^−^ and NO_*x*_ [56,57]. Since it is difficult to directly measure NO, nitrite or nitrate, which is a product of reactions with other reactive species, are mainly measured. Therefore, we evaluated the release of nitrite formed as a result of interaction between NO and water to investigate the regenerative power of LTP-treated S-A hydrogel [58]. Aligned with our previous study of a silk-fibrin membrane in skin flap wound healing model, the effect of LTP-treated S-A hydrogel through RONS including NO on wound healing could be verified by ECM deposition and neovascularization *in vitro* [59]. An *in vivo* rabbit model showed that LTP-treated S-A hydrogel increased the deposition and even distribution of collagen type I and fibronectin and normalized VF epithelium at the site of injury, which led to reduction of scar formation and recovery of viscoelasticity. Better functional restoration of VF vibration was observed in LTP-treated S-A hydrogel compared to S-A or alginate hydrogel, as measured by videokymography.

When an injury occurs in VFs, similar to other wound healing processes, inflammatory cytokines such as IL-1β, TNF-α, and cyclooxygenase type 2 (COX-2) drive the activation of tissue fibroblast [6]. Transforming growth factor-β (TGF-β), matrix metalloproteinases (MMP) and nuclear factor-κΒ (NF- κΒ) released by activated fibroblasts induces uncontrolled collagen production and fibrotic processes to result in a scar [2]. LTP contributed to wound healing of VF injury by activating VFF and decreasing inflammatory responses such as NF- κΒ and interleukin-6 (IL-6) [60]. Silk is also known to contribute to wound healing through anti-apoptotic, NF- κΒ, and mitogen-activated protein kinase (MAPK) pathways [61]. In this investigation, molecular pathways of wound healing, ECM synthesis and inflammatory response, were modulated by LTP in an *in vivo* model, as in other studies [62,63]. LTP-treated S-A hydrogels were maintained at 28 days after injection in an *in vivo* mouse model, demonstrating sustained efficacy with relatively slower degradation compared to other naturally-derived hydrogels such as those with fibrin, collagen, or gelatin. Sustained release of LTP from S-A hydrogel after injection into injured rabbit VF ameliorated wound healing in the remodeling phase, which takes place 2–3 weeks after injury [64]. LTP-treated S-A hydrogel, which showed controlled degradation and release of plasma effector due to crosslinking of silk, can be considered as a plausible injectable agent for wound healing.

Scar formation after VF injury (e.g. trauma, surgery, infection, and inflammation) causes increased stiffness and reduced mucosal vibration, leading to decreased voice function [65]. Injection laryngoplasty is the most common treatment option for VF injury [66], and the development of ideal injectable hydrogels for injection laryngoplasty has been diversely researched in the field of translational medicine (ECM component, stem cells, and bioactive factors) [[67], [68], [69], [70]]. However, no injectable hydrogel for VF injury has been accepted as a magic bullet since no treatments can fully restore the viscoelastic layer of the lamina propria and the vibration structures most important to phonation. For this reason, biomaterial for VF should consider viscoelasticity, because the unique collagen-rich subcutaneous structure called the lamina propria has viscoelasticity with a damping ratio (calculated by viscous shear modulus/elastic shear modulus) of approximately 0.3 [71]. In the rheology test, 60 ​s-HeO_2_-treated-LTP S-A hydrogel revealed a damping ratio of 0.3. LTP-controlled *in situ* silk crosslinking could be considered a successful strategy for injectable material due to adjustability of the unique viscoelasticity of the VF. An *in vivo* experiment verified the contribution of LTP-treated S-A hydrogel to VF injury. The LTP-treated S-A hydrogel group showed grossly acceptable mucosal wound healing on endoscopic image, histologic epithelial normalization, and functional symmetric VF amplitude in videokymography. This suggests that LTP-treated S-A hydrogel improved the wound healing process of injured VF.

In conclusion, LTP induced *in situ* crosslinking of injectable silk-based hydrogel, as confirmed by reduced gelation time without cytotoxicity, better mechanical properties, and enhanced wound healing in VF injury in both *in vitro*, and *in vivo* analyses. LTP-treated S-A hydrogel might be a promising injectable drug in VF injury, due to not only echo-friendly controlled *in situ* crosslinking but also increased regenerative potential through sustained release of plasma effector.

## Credit author statement

**Sungryeal Kim:** Conceptualization, Investigation, Validation, Data curation, Writing - original draft. **Hye-Young Lee**: Conceptualization, Methodology, Investigation, Visualization, Writing - original draft. **Hye Ran Lee**: Formal analysis, Validation, Writing - Review & Editing. **Ju Hyun Yun**: Formal analysis, Validation, Writing - Review & Editing. **Jeon Yeob Jang**: Methodology, Investigation, Writing - Review & Editing. **Yoo Seob Shin**: Conceptualization, Methodology, Writing - Review & Editing, Supervision, Funding acquisition, Project administration. **Chul-Ho Kim**: Writing - Review & Editing, Supervision, Project administration.

## Declaration of competing interest

The authors declare that they have no known competing financial interests or personal relationships that could have appeared to influence the work reported in this paper.
