# Supplementary material for: Liquid-type plasma-controlled in situ crosslinking of silk-alginate injectable gel displayed better bioactivities and mechanical properties
Source: Mater Today Bio. 2022 Jun 10;15:100321. doi: 10.1016/j.mtbio.2022.100321 (PMC9214807; doi:10.1016/j.mtbio.2022.100321)
Supplement: Multimedia component 2 [file mmc2.doc]

**Supporting Information**

**Liquid-type Plasma-Controlled In Situ Crosslinking of Silk-Alginate Injectable Gel Displayed Better Bioactivities and Mechanical Properties**

Sungryeal Kim^1,2^, Hye-Young Lee^3^, Hye Ran Lee^4^, Jeon Yeob Jang^3^, Ju Hyun Yun^3^, Yoo Seob Shin^3^*, and Chul-Ho Kim^3^

1. Department of Otolaryngology, College of Medicine, Inha University, Incheon, Korea

2. Department of Medical Sciences, Graduate School of Ajou University, Suwon, Korea

3. Department of Otolaryngology, School of Medicine, Ajou University, Suwon, Korea

4. Department of Otorhino-laryngology-Head and Neck Surgery, Catholic Kwandong University, College of Medicine, Incheon, Korea

**Address for Correspondence:**

^*^Yoo Seob Shin

Professor

Department of Otolaryngology,

Ajou University School of Medicine,

164 World-Cup Street, Yeongtong-gu, Suwon, 16499, Republic of Korea

Tel: +82-31-219-5262, Fax: +82-31-219-5264, E-mail: ysshinmd@ajou.ac.kr

**Content:**

**Supplementary table 1**

**Supplementary figures 1,2**

**Supplementary video 1:** A video showing the immediate gelation of LTP-treated S-A hydrogel as injected through syringe.

**Supplementary Table 1.** Specific concentration of component of hydrogel.

| **Solvent** | **Silk fibroin** | **Alginate** | **Weight of hydrogel** | **Calcium chloride** |
| --- | --- | --- | --- | --- |
| Distilled water | 8~10 wt% | 1 %(w/v) | 0.2 mL | 250mM |


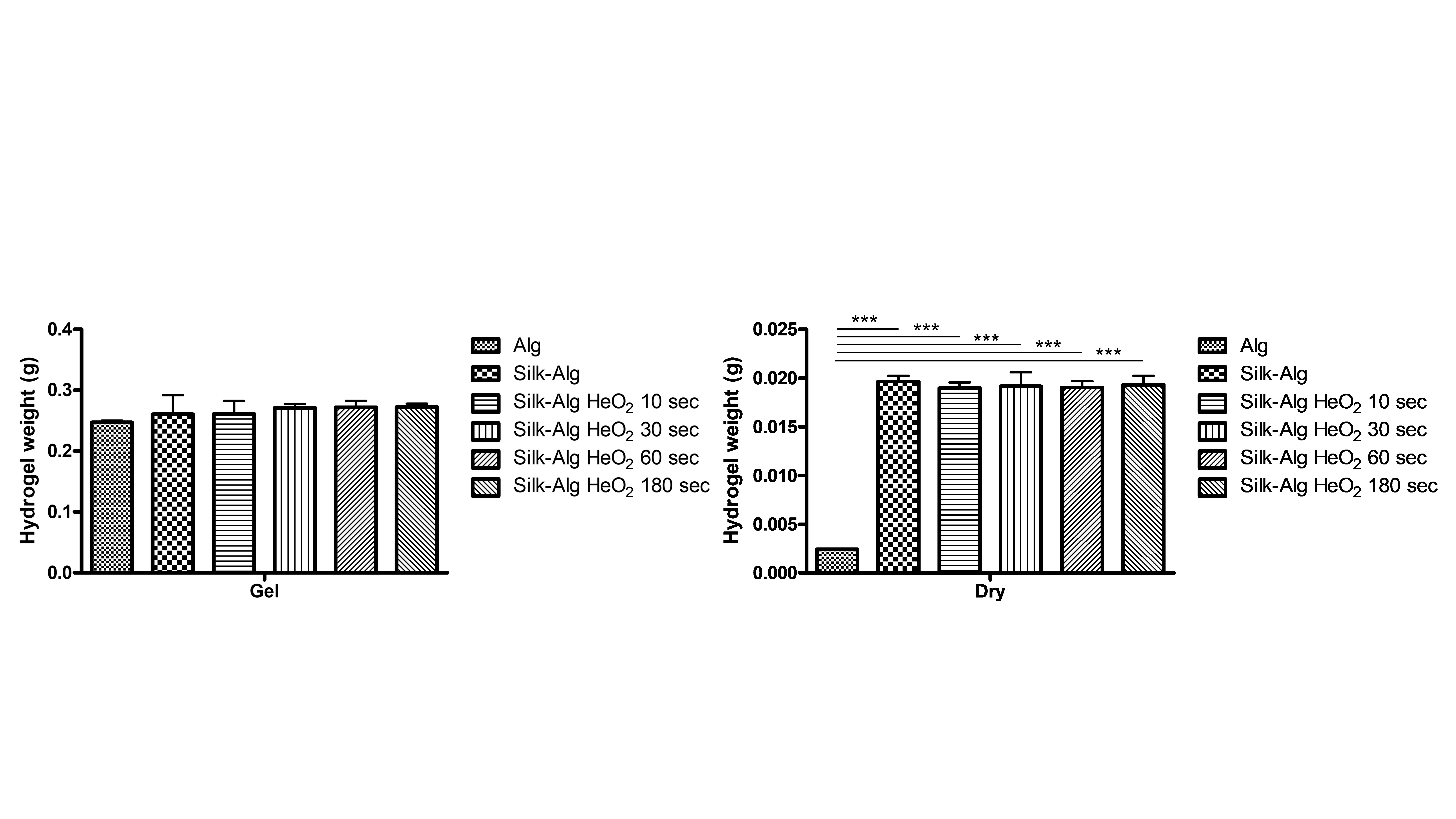


**Figure S1.** Weight comparison of hydrogels before and after freeze-drying.


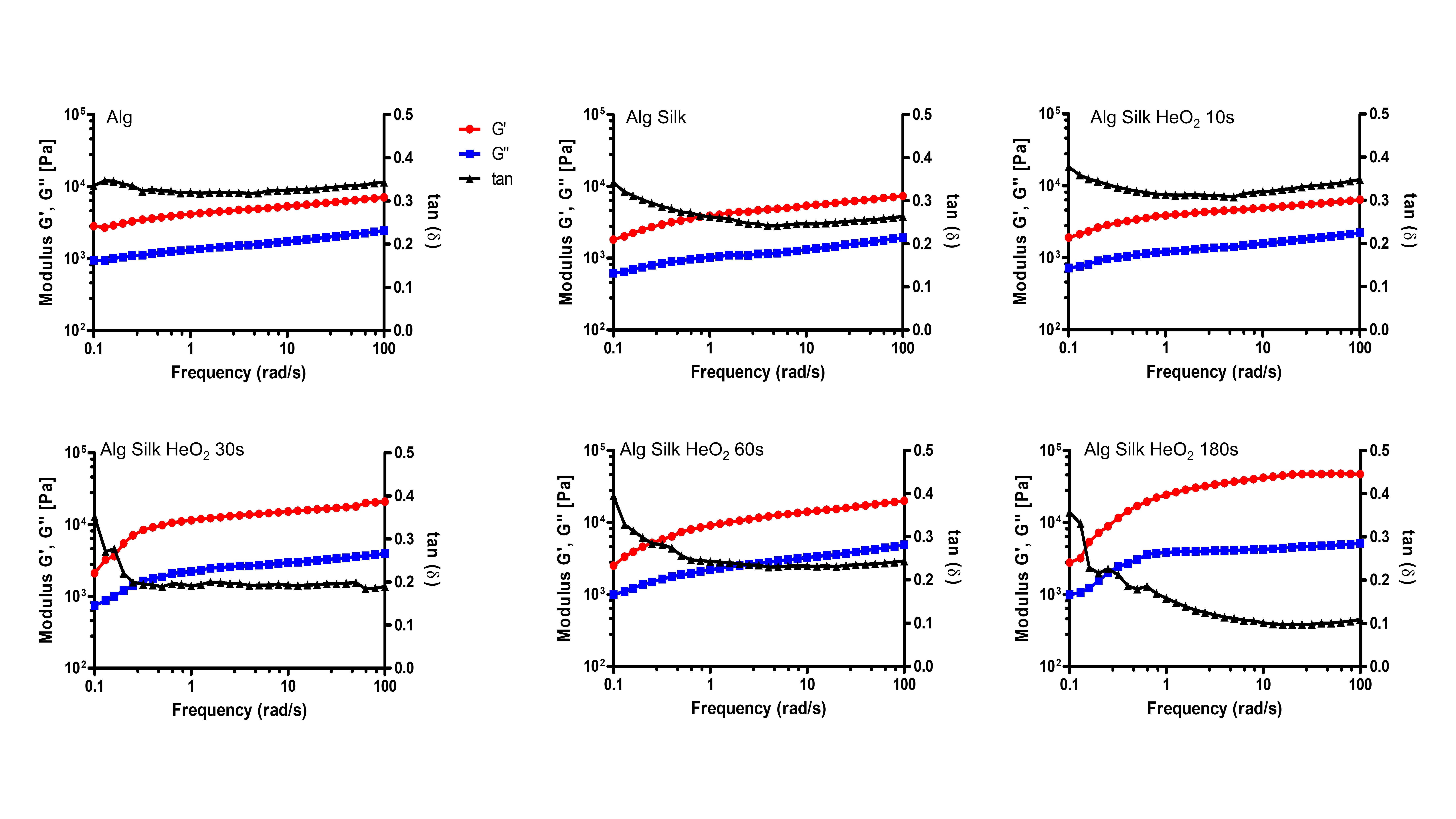


**Figure S2.** Storage (G’) and loss (G’’) modules of each hydrogel.
